# Male Breast Cancer: Growing Insights and Continuing Challenges

**DOI:** 10.1002/cam4.71673

**Published:** 2026-03-03

**Authors:** Sahar Iftikhar, Elissa Burr, Amal Freigoun, Theo Nearney, Christelle Q. Kanda, Hazel Robinson, Caroline Vermeren, Udana Wickramaratne, Matthew Everest, Luca Foley, Valerie Speirs

**Affiliations:** ^1^ School of Medicine, Medical Sciences and Nutrition University of Aberdeen Aberdeen Scotland, UK

**Keywords:** breast cancer, epidemiology, men, stigma

## Abstract

**Background:**

Breast cancer (BC) in men accounts for less than 1% of all BC diagnoses worldwide. Despite its low incidence, the number of men being diagnosed is increasing. Historically perceived as a predominantly female disease, BC in men has received comparatively limited attention.

**Aim:**

This review aims to examine how BC manifests in men and explored the impact of stigma and awareness on diagnosis and prognosis.

**Methods:**

A narrative review of current literature was undertaken, including epidemiological studies, clinical research and psychosocial analyses relating to male BC.

**Results:**

Male BC shares several pathological and molecular features with female BC, but notable differences exist in presentation, tumour biology and treatment considerations. Men are more likely to present at a later stage of disease, often due to low awareness and misconception that BC affects only women. Stigma and limited targeted education further contribute to delayed medical consultation.

**Discussion:**

Although understanding of male BC has improved, challenges remain in early detection and awareness. The gendered perception of BC may discourage men from seeking timely medical advice, leading to more advanced disease at diagnosis and potentially poorer outcomes.

**Conclusion:**

Addressing stigma, increasing public and professional education and promoting clinical research inclusive of men are essential to improving timely diagnosis and outcomes.

## 1 Introduction

“I never thought it could happen to me.” These words often resound with men facing a breast cancer (BC) diagnosis, evoking emotions of shock, surprise, distress, disbelief and embarrassment alongside dealing with the psychological stress of being diagnosed with what many still see as a ‘woman's disease’ [1, 2, 3]. This is perhaps not surprising as BC has been associated with women since its discovery, with particular emphasis during the 18th and 19th century coinciding with advancements in surgical interventions. As these surgeries were primarily performed on women this further reinforced the narrative that breast cancer exclusively occurred in women [4]. Moreover, by the mid‐19th century there was an overwhelming outlook that cancer in general mostly affected women, largely because BC was the most visible and more accurately diagnosed form of the disease at that time. Indeed, a contemporaneous standard medical textbook stated: “There is no fact in the history of cancer more absolutely demonstrated than the influence exercised by sex on its development,” further reinforcing this idea by stating that women were affected by cancer at a rate of “2 and three quarters” greater than men [5].

We now know that this is not true, and that BC can affect men. However, it accounts for only 1% of all breast cancer cases and 0.2% of male deaths of any kind of cancer. Indeed, of 31 cancers which displayed sex disparities, BC showed the biggest divergence [6]. Nevertheless, the association of BC with women prevails, in part because of successful public health campaigns designed to raise BC awareness. Many of these campaigns are often saturated in shades of pink and reinforced with slogans exuding femininity. Inadvertently, this alienates men with BC, creating potential barriers for men in recognising symptoms, seeking support, and further stigmatising this diagnosis.

There have been a plethora of reviews discussing general clinicopathological features of male BC, with some examples provided [7, 8, 9, 10]. Hence the aim of this study was to take a more focused approach to discuss how a disease with such deeply rooted connections to women manifests in men and how it differs between sexes. We also consider how we can raise awareness in men so that they can recognise the signs of BC and seek earlier interventions.

## 2 Anatomy of the Male and Female Breast

**FIGURE 1:**
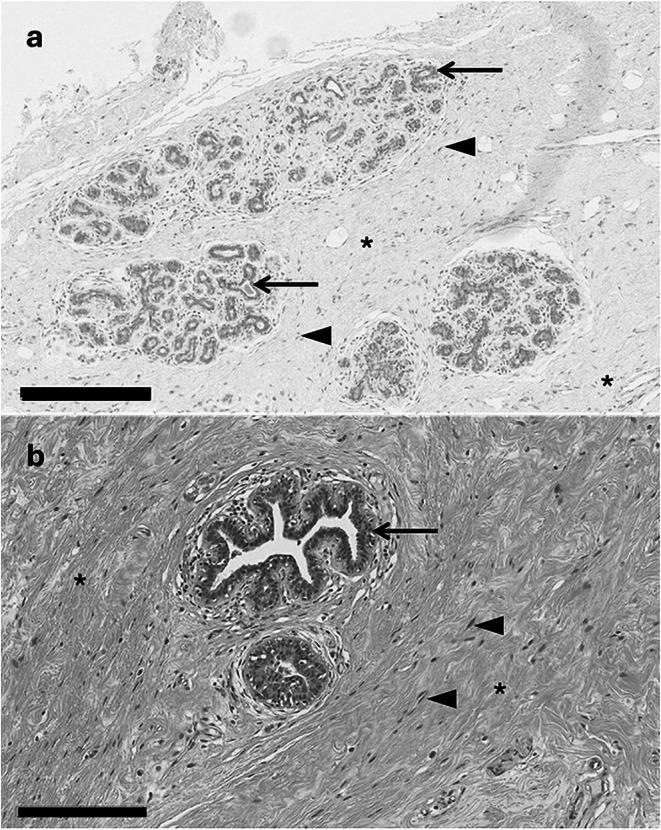
Histological appearance of normal breast male and female mammary gland. In females (a), breast ducts (arrows) are organised into terminal ductal lobular units (dotted lines) while the male mammary gland (b) is simpler with scattered ducts (arrows). Ducts and lobules are set within a collagen‐rich stroma (asterisks) which contains fibroblasts (arrowheads). Scale bar = 300 μ (female), 200 μ (male). Tissue images were generated from anonymised male and female breast tissue samples donated with ethical approval to the Leeds Breast Tissue Bank (15/YH/0025) whose samples now reside in the Breast Cancer Now Biobank (23/EE/0229).

Histological images of male and female breast tissue are shown in Figure 1. Macroscopically, the female breast is composed of glandular tissue of around 15–20 lobes. Each lobe contains lobules and lactiferous ducts which meet at the nipple. The terminal ductal lobular unit (TDLU) is the functional unit of the breast, comprising epithelial cell‐lined ducts with myoepithelial cells surrounding these. TDLUs are the site where most breast cancers originate [11]. Fatty connective tissue surrounds mammary glands and helps to stabilise them. Additionally, collagen‐rich stroma works to anchor the dermis and underlying pectoral fascia as well as divide the breast's secretory lobules. The stroma also plays a role in cancer invasion and progression. Anatomically, male breast tissue shares key structural similarities such as ducts, nipples, and areolas. However, breast ducts are scattered and sparse compared to their abundance in female breast tissue.

## 3 Development and Classification of Male BC

BC commonly arises from the breast ducts, with invasive ductal carcinoma (IDC) of no special type being most common in both sexes. IDC is especially prevalent in men while DCIS, triple negative, and HER2‐positive BC are uncommon [12]. Oestrogen receptor (ER) is a key prognostic and predictive biomarker in BC [13]. In a retrospective analysis of 1483 male patients with BC, ER positivity was reported in > 90% of cases [14]. This is at odds with female BC where ER is expressed in around two thirds of all cases. ER‐positive tumours have a better prognosis as they are suitable for adjuvant endocrine therapy. In women, this includes selective oestrogen receptor modulators (SERMs), commonly tamoxifen and aromatase inhibitors such as anastrozole, which are mainstays in management. Because of the high proportion of ER expression in men, endocrine therapy has been the mainstay of treatment for men with BC, with tamoxifen the recommended standard of care for men in line with the latest ASCO guidelines [15].

ER‐positive female BC is further categorised into luminal A and luminal B. The former comprises about 60% and is responsive to adjuvant endocrine therapy. Luminal B makes up the remainder and can sometimes be refractory to endocrine therapy, often requiring treatment with chemotherapy and/or biological therapies [16]. Outcomes can also be worse for this subtype. Based on the molecular classifications used in female BC, data from the International Male Breast Cancer Program identified that of 1483 male BCs analysed, 42% were Luminal‐A and 49% Luminal‐B [14]. However, transcriptomic studies have allowed more refined classification of male BC, showing that this can be split into male complex and simplex subtypes [17, 18]. Additional transcriptomics analysis is starting to show greater divergence between male and female BC, including enrichment in mutations affecting DNA repair‐related genes in male BC [19, 20]. A systematic review analysed genomic, transcriptomic, proteomic, epigenetic, and phenotypic biomarkers in male BC, highlighting not only its complexity and heterogeneity but also notable dissimilarity with female BC [9]. Recently, a machine learning approach has shown that models trained to detect ER in female BC do not generalise to BC in men, further pointing towards biological differences between the sexes [21].

## 4 Incidence and Outcome of Male BC

**FIGURE 2:**
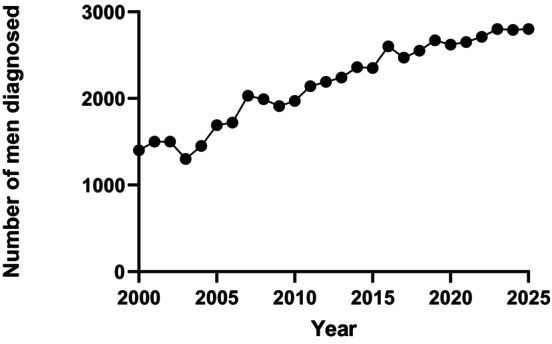
Trends of incidence in male breast cancer in the United States from 2000 to 2025. Figure created using data extracted from the CA: A Cancer Journal for Clinicians, annual Cancer Statistics compiled from data generated by the American Cancer Society, based on the original figure by Tay [24].

The numbers of male BC being diagnosed is rising, supported from various sources. Annual statistics published by the American Cancer Society show that in the United States, the numbers of new cases of male BC have risen from 1400 in 2000 [22] to 2800 in 2025 [23]. This is shown in Figure 2.

Data obtained from the Global Burden of Disease database for 123 countries showed that the incidence rose from 8500 in 1990 to 23,100 in 2017, an increase of over 200% [25]. Similar increases have been reported in the UK [26, 27]. Lifestyle factors may be responsible, but this is challenging to define conclusively. Interestingly, the numbers of men being diagnosed with BC rival increasing obesity [28]. Men typically delay presenting to their GP for 6–9 months after becoming symptomatic [29]. Symptoms are sometimes dismissed as benign conditions such as gynecomastia, which has no conclusive evidence linking it to BC, which may contribute to diagnostic delays.

Nearly half of all men have axillary lymph node involvement at diagnosis [30]. Analysis from the Surveillance, Epidemiology, and End Results (SEER) database of 2054 men with a primary breast cancer diagnosis from 2005 to 2010 showed reduced 5‐year survival for male patients [31]. This is supported by more recent SEER analysis of 16,025 men diagnosed with BC between 2004 and 2014, where 5‐year overall survival rates were 77.6% for men versus 86.4% for women [32]. This difference remained even after adjusting for clinical characteristics, treatment factors, age, race/ethnicity, and access to care, demonstrating that sex was a significant factor associated with overall mortality [32]. The same study showed that overall mortality rates for men were 19% higher than those for women [32]. Furthermore, men diagnosed with early stage ER‐positive BC face a risk of recurrence and death which persists for 20 years after diagnosis [33]. BC in men is thought to resemble late‐onset BC in women [34] hence greater understanding of why outcomes are worse in men with BC need to be elucidated. Later diagnosis, when treatment is less likely to be effective, may well contribute, but there may be other factors at play. This is being addressed through large, centralised collections of male BC established through multi‐site collaboration [35, 36].

## 5 Screening and Genetics

Although more men are being diagnosed with BC, it still accounts for less than 1% of BC cases making it hard to justify screening programmes like those for women. Nevertheless, the utility of screening men at higher risk of BC e.g., those with loss of function mutations in the DNA damage response genes *BRCA1* and, in particular, *BRCA2* where some 10% of cases are attributable to inherited mutations of the latter [37, 38] has been investigated. This found that mammographic screening detected 4.9 cancers per 1000 examinations, comparable to the detection rate in women at average risk and demonstrating the value for screening mammography in high‐risk male patients [39].

Studies aiming to identify genetic variations that increase men's risk of developing BC have been conducted. Mutations in *CHEK2* and *PALB2* DNA‐repair are associated with male BC [40, 41]. Single nucleotide polymorphisms in *RAD51B* at 14q24 and in *TOX3* at 16q12.1 conferred greater risk of BC in men than women [42]. A genome‐wide association study identified 3 novel susceptibility loci, two mapping to 6q25.1 and another to 11q13.3, that were unique to male BC [43]. Finally, there is intriguing but unexplained evidence showing that BC risk is increased two‐fold in infertile men [44].

## 6 Clinical Trials

Historically, men were excluded from BC clinical trials, which focused on women only. Consequently, treatment recommendations for men are based on data extrapolated from trials in women. This is an issue in BC because men are generally under‐represented in clinical trials, meaning that treatments are not wholly evidence based. Analysis of 131 randomised BC clinical trials revealed that male patients represented only 0.087% of the total study population [45], a statistic that is lower than the purported 1% of men that are diagnosed with BC worldwide [7].

Early attempts to establish specific trials for men had good intentions but frequently faced recruitment challenges resulting in early termination. The International Male Breast Cancer Programme planned a trial initially but instead focused on establishing successful research networks [35]. More recently, a multicentre, randomised phase 2 clinical trial of 56 men with ER‐positive BC recruited from 24 breast units across Germany completed successfully [46]. This trial examined the impact of 6 months of tamoxifen alone or tamoxifen plus gonadotropin‐releasing hormone analogue (GnRHa) or aromatase inhibitor (AI) plus GnRHa on oestrogen levels [46]. Data showed that AI or tamoxifen plus GnRHa vs. tamoxifen alone decreased systemic oestrogen. However, this resulted in impaired sexual function and quality of life. The ongoing ETHAN clinical trial has been established to compare AIs, gonadal suppression, and cyclin‐dependent kinase 4/6 (CDK4/6) inhibitors in men 60 men with BC in the United States [47]. These studies indicate that if small and focused, male‐specific BC trials can be achieved.

Furthermore, exclusion criteria for some BC clinical trials have been relaxed meaning that men can now be enrolled [48]. MonarchE, a randomised phase III trial, showed the combination of adjuvant CDK4/6 inhibitor abemaciclib and endocrine therapy had a lasting and improved effect compared to endocrine therapy alone, amplifying progressive disease‐free and relapse‐free survival. This advantage persisted with an absolute increase at 4 years [49]. MONALEESA‐3, a phase III placebo‐controlled trial, included both male and female patients and explored the effects of combining ribociclib with fulvestrant [50]. This displayed an improvement in overall survival compared to fulvestrant alone, thus demonstrating the efficacy of CDK4/6 inhibitors in sustaining progression‐free survival. The effects of fulvestrant with a P13K inhibitor, alpelisib, in *PIK3CA*‐mutated breast cancer have also been studied in men [51]. While this significantly lengthened progression‐free survival, pronounced side effects were reported, including hyperglycaemia (remedied by metformin) and diarrhoea.

## 7 Stigmatisation

With BC the most common cancer in females worldwide it gains significantly more publicity and research than male BC. This is due in part to advocacy which has increased public awareness internationally and considerable resources have been channelled into research addressing disease aetiology, early detection, treatment and more recently, preventative strategies.

These actions serve to reinforce the misconception that BC is exclusively a women's disease. Not only do men with BC have to deal with their diagnosis and its management, but being diagnosed with a disease in a part of the body not readily associated with men may evoke questions about their masculinity. There is also the psychosocial impact surrounding body image, particularly after surgery [52, 53].

Studies exploring the stigmatisation of male patients found that some of this happens within the cancer care system itself [54, 55]. One patient interviewed claimed he was laughed at and told he didn't belong there when he presented at a breast cancer care centre. Another patient claimed he was mistakenly called by a female pronoun while in the waiting room. Similar instances were experienced by multiple patients in the study, furthering the feelings of exclusion. These observations serve to remind us that there is often an underlying assumption that a man attending a breast clinic is there to support his partner rather than being there as a BC patient [56].

Cultural differences can result in challenges in breast cancer management in men. An Indian study explored the knowledge of male BC among 128 men who had heard of the condition [57]. Just over half of the men did not know that changes to the nipple was a potential sign of BC. Moreover, 60.9% were unaware that having family members, male or female, with BC increased their chance of having it. Over 90% of the men had no understanding of how to perform a self‐breast examination, and a third admitted to being embarrassed at the idea of a BC diagnosis. The study concluded there was inadequate awareness and understanding around the disease among the public, as well as feelings of shame at having a ‘woman's disease’ [57].

A case report described a Tanzanian male presenting with a 24 × 24 cm breast tumour that occupied the entire breast who refused all forms of medical treatment, instead opting for traditional medicines [58]. Another case report described an Afghan male of Asian ethnic origin who was an asylum seeker in the UK and presented with a 30 × 13 mm mass which was subsequently diagnosed as BC [59]. He could not accept a BC diagnosis and refused all forms of treatment initially. It was only after speaking to a breast care nurse, who was fluent in the patients' native tongue and knowledgeable of the Afghan culture that he started to come to terms with his diagnosis and accept treatment [59]. These case reports highlight clinical obstacles that can be encountered when managing a male BC in ethnic minorities where language and cultural beliefs can add additional layers of complexity.

The stigma of being a ‘woman's disease’ not only has an impact on the emotional wellbeing of male BC patients but also affects their diagnosis and care. Because many are unaware that males can get it, numerous cases have occurred where men presenting with red flag symptoms have had missed or late diagnosis, which greatly influences the prognosis. In one case, a young man in his thirties was diagnosed with invasive ductal carcinoma after presenting with a lump in his left breast which he had noticed 6 months prior [60]. Unfortunately, 3 years after his mastectomy the disease had metastasised extensively, reaching an advanced stage beyond treatment and ultimately leading to his death. Had the patient been more aware of the possibility of BC and sought medical advice when he first noticed the lump, there may have been a better outcome. This is one of many cases where delayed diagnosis decreases the chance of a successful treatment due to significant deterioration in the patient's condition.

An often‐overlooked consequence of the stereotype is the change in body image that many men face after surgery [3]. This can have a negative impact on self‐perception as masculinity is often associated with the chest, and scarring after surgery can act as a reminder of emasculation [2, 3]. Some have turned this into a positive noting that their surgical scar can serve as a conversation piece [55]. Adverse side effects can arise from taking tamoxifen, a drug which is well researched in women, but less so in men. Men taking tamoxifen often report feelings of anxiety, and sexual dysfunction is a common adverse reaction [61]. This can add to the psychological burden of the diagnosis and potentially cause intimacy issues and affect their emotional well‐being [3]. Side‐effect profiles of tamoxifen may reflect innate sex differences in the endocrinology and drug metabolisms between men and women [61].

## 8 Signposting

**FIGURE 3:**
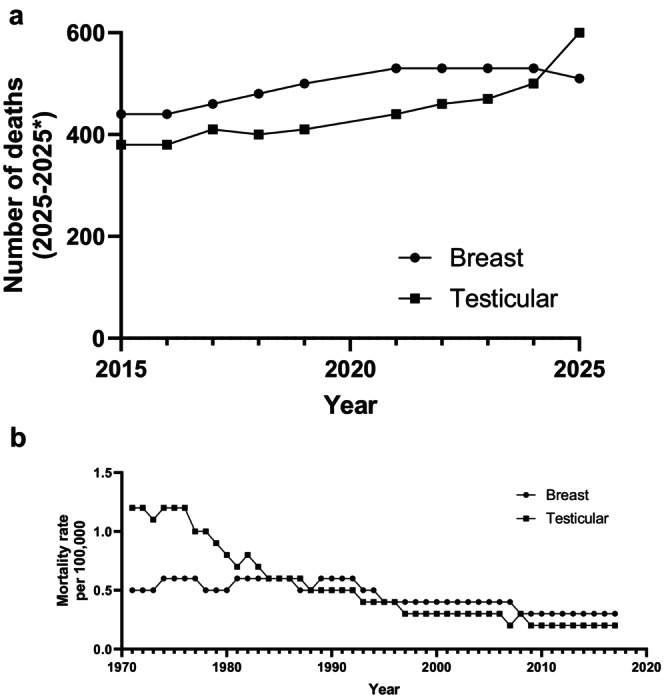
Comparison of mortality in men with breast and testicular cancer. (a) Generated from data extracted from the CA: A Cancer Journal for Clinicians, annual ‘Cancer Statistics’ compiled from data generated by the American Cancer Society. *Data not available for 2020. (b) Generated from Cancer Research UKs Cancer Statistics for the UK data 1990–2017 [64].

Reduced public knowledge of BC in men is unsurprising, with resources and publicity regarding the disease predominantly catered towards women. This view was corroborated in a questionnaire distributed to 411 patients (270 female, 141 male) where 61% did not believe that men could get BC [62]. In England, the Be Clear on Cancer campaign has been running since 2010 with evidence of positive impact on cancer diagnosis; patients have sought help earlier and there has been increased referral patterns by general practitioners [63]. These types of public health campaigns are excellent, but BC needs to be on men's radar for them to be aware of possible BC symptoms. Remarkably, mortality rates for male BC exceed those of testicular cancer (Figure 3), yet there is much greater awareness of this with April designated Testicular Cancer Awareness Month.

But this is starting to change. The Male Breast Cancer Global Alliance [65] advocates for all men diagnosed with BC to increase awareness. It brings together men with BC, clinicians, and scientists from around the world to accelerate research, clinical trials, and treatments in male BC. The Men's Virtual Meetup [66] provides a safe online forum where men diagnosed with BC can interact and engage with others. In 2021, a storyline about male BC was introduced into the UK soap opera EastEnders [67]. Interestingly, this was a recommendation from a focus group of men with BC reported in a study published nearly 20 years ago [1]. Another recommendation from that study was to display posters about BC in men in places where men typically frequent [1]. While this has not yet been actioned, it could be incorporated into Movember campaigns [68] aimed to initiate conversations about men's health to raise public awareness of the health risks men face, including BC. Many cancer charities now include male‐specific messaging to increase awareness about BC. Macmillan Cancer Support now produces literature “Understanding Breast Cancer in Men” designed specifically for men [69] and male‐specific information booklets devoid of shades of pink have been introduced by the BC research and support charity, Breast Cancer Now [70]. These are all positive steps which should help reduce stigmatisation and raise awareness in men about potential symptoms of BC, encouraging earlier presentation and diagnosis, resulting in better outcomes.

## 9 Conclusion

Over the last couple of decades, the narrative on male BC has begun to change gradually. Research on male BC specifically has progressed significantly, and our scientific understanding continues to improve. However, the rarity of a BC diagnosis in men, combined with the societal stigmas and labels which have been associated with this historically, can put men with symptoms of the disease in an unusual situation. They often delay seeking medical attention due to embarrassment and societal misconceptions, putting them at risk of a poorer prognosis. While positive steps have been taken towards signposting the disease, more work is needed to improve patient experience and outcomes. This will continue to alter society's views and stigmas, creating an environment where men feel comfortable discussing their symptoms, seeking medical advice, and understanding that BC is not just a ‘woman's disease’. By challenging outdated stereotypes, promoting education, and advocating for personalised medicine through knowledge gained from research, we can move towards a future where early diagnosis and effective treatments prevail. These are important steps towards breaking the stigma of BC in men ensuring that men affected receive early diagnosis, personalised care, and the practical and emotional support that they need.

## Author Contributions

**S.I**.: writing, review and editing, methodology, investigation, formal analysis, data curation. **E.B**.: writing, review and editing, methodology, investigation, formal analysis, data curation. **A.F.:** writing, review and editing, methodology, investigation, formal analysis, data curation. **T.N**.: writing, review and editing, methodology, investigation, formal analysis, data curation. **C.Q.K**.: writing, review and editing, methodology, investigation, formal analysis, data curation. **H.R**.: writing, review and editing, methodology, investigation, formal analysis, data curation. **C.V**.: writing, review and editing, methodology, investigation, formal analysis, data curation. **U.W**.: writing, review and editing, methodology, investigation, formal analysis, data curation. **M.E**.: writing, review and editing, methodology, investigation, formal analysis, data curation. **L.F**.: writing, review and editing, methodology, investigation, formal analysis, data curation. **V.S**.: writing, review and editing, visualisation, supervision, project administration, methodology, investigation, formal analysis, data curation, resources, conceptualisation.

## Funding

The authors have nothing to report.

## Ethics Statement

The tissue images included in Figure 1 were generated from anonymised male and female breast tissue samples donated with ethical approval to the Leeds Breast Tissue Bank (REC 15/YH/0025), whose samples now reside in the Breast Cancer Now Biobank (REC 23/EE/0229). As both samples were provided with full anonymisation, the donors cannot be identified meaning that written informed patient consent was not required.

## Consent

The authors have nothing to report.

## Conflicts of Interest

The authors declare no conflicts of interest.

## Data Availability

Data sharing not applicable to this article as no datasets were generated or analysed during the current study.
